# A Systems Map of the Challenges of Climate Communication

**DOI:** 10.1080/10810730.2024.2361842

**Published:** 2024-06-06

**Authors:** JESSIE HENEGHAN, DANIELLE C. JOHN, SARAH M. BARTSCH, RACHAEL PILTCH-LOEB, CHRISTINE GILBERT, DAN KASS, KEVIN L. CHIN, ALEXIS DIBBS, TEJ D. SHAH, KELLY J. O’SHEA, SHERYL A. SCANNELL, MARIE F. MARTINEZ, BRUCE Y. LEE

**Affiliations:** 1 Center for Advanced Technology and Communication in Health (CATCH), CUNY Graduate School of Public Health and Health Policy, New York City, New York, USA; 2 Public Health Informatics, Computational, and Operations Research (PHICOR), CUNY Graduate School of Public Health and Health Policy, New York City, New York, USA; 3 Artificial Intelligence, Modeling, and Informatics, for Nutrition Guidance and Systems (AIMINGS) Center, CUNY Graduate School of Public Health and Health Policy, New York City, USA; 4 Pandemic Response Institute, New York City, New York, USA; 5 Environmental, Occupational, and Geospatial Health Sciences, City University of New York Graduate School of Public Health and Health Policy, New York City, New York, USA; 6 School of Communication & Journalism, Stony Brook University, Stony Brook, New York, USA; 7 Alan Alda Center for Communicating Science, Stony Brook University, Stony Brook, New York, USA; 8 School of Marine and Atmospheric Sciences, Stony Brook University, Stony Brook, New York, USA; 9 Vital Strategies, New York, New York, USA

## Abstract

Over the past sixty years, scientists have been warning about climate change and its impacts on human health, but evidence suggests that many may not be heeding these concerns. This raises the question of whether new communication approaches are needed to overcome the unique challenges of communicating what people can do to slow or reverse climate change. To better elucidate the challenges of communicating about the links between human activity, climate change and its effects, and identify potential solutions, we developed a systems map of the factors and processes involved based on systems mapping sessions with climate change and communication experts. The systems map revealed 27 communication challenges such as “Limited information on how individual actions contribute to collective human activity,” “Limited information on how present activity leads to long-term effects,” and “Difficult to represent and communicate complex relationships.” The systems map also revealed several themes among the identified challenges that exist in communicating about climate change, including a lack of available data and integrated databases, climate change disciplines working in silos, a need for a lexicon that is easily understood by the public, and the need for new communication strategies to describe processes that take time to manifest.

Over the past sixty years (Bell, 2021), scientists have been warning about climate change and its impacts on human health, but evidence suggests that many may not be heeding these concerns in the absence of substantial shifts to public opinion, policy, and human activity. For example, the United Nations (UN), Environmental Defense Fund (EDF), and Greenpeace have issued warnings and calls to action for governments and businesses to do more about climate change (Greenpeace, 2024; Kallhauge, 2023; United Nations, n.d.). Yet various polls have shown that many people are not willing to make changes that may help mitigate climate change and its impacts. For example, only 31% of Americans support a transition to fully renewable energy sources, and 14% do not even believe that climate change exists (Pasquini et al., 2023; Tyson et al., 2023; Weise, 2023). Global surveys demonstrate that climate change is widely perceived as an urgent issue, but also suggest that the expectation of collective responsibility (e.g., most countries adopt policies to combat climate change) may inhibit public demand for local actions (Dabla-Norris et al., 2023). This raises the question of whether new communication approaches are needed to overcome the unique challenges of communicating the impacts of climate change.

A major hurdle is that the processes involved in how human activity leads to climate change and how climate change then leads to changes in the environment and atmosphere and subsequent effects on human health comprise a complex system. Systems mapping is an established method to help people better understand complex systems (Barbrook-Johnson & Penn, 2021; Cox et al., 2021; Mabry et al., 2010; Pronk et al., 2023; Scott et al., 2016; van den Akker et al., 2023). Participatory systems mapping involves bringing stakeholders with different expertise together to collectively construct a “mental map” of a system, where they are asked to apply their knowledge of the mechanisms and relationships of the wider system to build the map together, working to uncover the “why” and “how” different events and processes come about (Ackermann & Alexander, 2016). This visual representation of the system elicits a richer, more nuanced, and holistic understanding of its complexity, including where feedback loops and leverage points may exist, and what may be needed to intervene in the system. This also helps build a narrative and generate new questions for a particular issue (Barbrook-Johnson & Penn, 2021). For example, one systems map shows the steps involved in vaccination and its associated costs and health benefits in order to identify the mechanisms that, when interrupted or delayed, may make getting vaccinated more difficult for hard-to-reach populations. This systems map helped to better elucidate where weak points may exist in the vaccination process, identify gaps in our understanding of the economics of vaccination, and help inform targeted interventions and policies to increase vaccination coverage in such populations (Cox et al., 2021). Therefore, we developed a systems map in order to better elucidate the challenges of communicating about the links between human activity, climate change and its effects, and identify potential solutions.

## Methods

### What Is a Systems Map?

A systems map is a diagram that visually represents all the components of a system and how they may interact with and affect one another (Mabry et al., 2022). Systems maps have been used to elucidate the systems involved with obesity, vaccination, and a number of other health and public health related issues (Barbrook-Johnson & Penn, 2021; Cox et al., 2021; Lee, Bartsch, et al., 2017; Mabry et al., 2010; Pronk et al., 2023; Scott et al., 2016; van den Akker et al., 2023). Participatory systems mapping is used when the emphasis is on stakeholder engagement and ownership, and the purpose is to include as much complexity as possible to fully capture the systems of interest (Barbrook-Johnson & Penn, 2021). Such an approach can be easy to use, practical, and flexible, while at the same time, can also provide rich insights accounting for the complexity of the system (Barbrook-Johnson & Penn, 2021; Bertscher et al., 2024). Since we aimed to be stakeholder-driven and generate an array of insights, we developed a systems map rather than using other methods such as focus groups, which can be less interactive and would not result in a diagram, as focus groups typically do not generate an outcome together (Stickdorn et al., 2018).

Table 1 shows the conventions [derived from the Unified Modeling Language (UML) (Lucidchart, n.d.-a, n.d.-b)] that we used to develop this systems map. UML was used in the absence of other standard systems mapping conventions, as this has been used to describe processes and workflows in other fields such as business and healthcare (Kumarapeli et al., 2007; Luzi et al., 2019; Mincarone et al., 2018; Pecoraro & Luzi, 2022; Vasilakis et al., 2008). The convention consists of shapes that represent different components of the system (e.g., causes, effects, and factors that affect each) and arrows showing relationships and the direction of the cause and effects between these components. Additionally, we intentionally used reader-friendly language, rather than more technical systems modeling language, throughout the map so that it could be readily interpreted by a variety of audiences. This is especially important as climate change spans various disciplines and audiences (e.g., communications, climate scientists, researchers, general public).

### The Systems Mapping Process

We used participatory systems mapping methods to develop the systems map (Barbrook-Johnson & Penn, 2022). We first decided on the aim of the map and then defined the system’s boundaries. This began with identifying the question of interest, “What are the unique challenges in communicating to the public about the causes and effects of climate change, including the impacts to human health?” To answer this question, we decided to develop a systems map to better understand the complex relationships between the causes and effects of climate change, impacts to human health, and communication challenges that arise from these factors. We collectively agreed to bound the systems map by focusing on developing a general representation of the processes of climate change, and then mapping the communication challenges that arise from communicating about these processes. Next, we used our academic and professional networks to identify climate change and climate communication experts and then contacted each expert via e-mail to request their participation in the systems mapping process. We chose the experts based on their demonstrated knowledge, experience and relevant research in the areas of climate change, environmental health, climate change communication, and public health communication, which we identified as critical perspectives for developing the map. For example, one participant had expertise in pollution and human health, including the role of climate change, which was an important perspective for representing the core causes and effects of climate change in the map. Another participant had expertise in health emergency preparedness and response and how to engage the public during public health emergencies, including climate change, which was a critical perspective needed to add communication challenges to the map. The other two participants had expertise in how individual characteristics and media habits influence how people respond to risks and challenges related to climate change as well as environmental health and environmental disease prevention with a focus on policy, research, risk assessment, and communication, respectively, which added additional perspectives important for identifying the various challenges that arise from communicating the processes and impacts of climate change. We then conducted one-on-one virtual sessions over Zoom with each of the four experts that agreed to participate to collect their feedback and collaboratively build the map in two portions. We conducted one-on-one sessions given restricted schedules among the experts and to avoid “group-think.” Multiple one-on-one sessions with various experts led to rich conversations and the map developing iteratively over time. First, we focused on the processes involved in climate change and its impacts on human health. Then, we identified the challenges faced when communicating with the public about these different processes involved in climate change and its impacts on human health.

### The Processes of Climate Change Portion of the Systems Map

We first sought to establish a very general representation of the processes and components involved in climate change, starting with individual and collective human activity that contributes to climate change that impacts different parts of the environment and ending with the human health, societal, and economic effects that accrue as a result. The goal was not to represent every single mechanism in significant detail, but instead to create a framework visualizing the major causes and effects of climate change. This framework serves as a base to then identify the unique challenges that make communicating the causes and effects of climate change difficult, as well as the impact these challenges have on individual behavior and decision making related to climate change.

Our team developed an initial version of *The Processes of Climate Change* portion of the map and then held systems mapping sessions with experts to collect their feedback and refine this version. We began each systems mapping session with a brief explanation of the goal of our map and a walkthrough of the initial map. We prompted feedback on this portion by asking guided questions about the overall system of the major causes and effects of climate change that result in human health effects. Examples of such questions include:

1. “Are the impacts of human activity on climate change clear and relevant, and are we missing any key ones?”
2. “Are the direct environmental impacts of climate change clear and relevant, and are we missing any major ones?”
3. “Are there any intermediary steps that we have not accounted for?”
4. “Should we include specific health conditions (e.g., respiratory, weight-related) to represent human health effects (while avoiding drawing connections that are not definitive)?”

During the sessions, one team member led the discussion, another screenshared the map and made real-time edits based on the expert feedback, and a third took notes of the discussion. With real-time edits, experts were able to review the changes to ensure their input was correctly incorporated and provided a chance to further refine. When the expert was unable to schedule a virtual session, we accepted written feedback and responses to the guided questions via e-mail.

### The Challenges of Climate Change Communication Portion of the Systems Map

Using *The Processes of Climate Change* part of the map as a base, we then sought to identify the specific elements that make it challenging to communicate each factor/process involved in climate change. The objective was not to represent the quality of the climate information itself or people’s biases that may impact their acceptance of the information, but rather to represent the challenges of communicating the factors/processes that lead to climate change and its ultimate impacts on the environment/atmosphere and human health.

We conducted subsequent feedback sessions with the experts on this portion of the map by prompting feedback with guided questions about what makes communicating each factor/process and their relationships in *The Processes of Climate Change* portion of the map challenging. Examples of such questions include:

1. “In what ways is communicating the link between human activity and climate change difficult?”
2. “Where do you see people most often not understanding climate information, and why do you think that is the case?”
3. “What impacts of climate change seem unclear to people and why?”

Similar to the mapping sessions described above, we made real-time edits. Additionally, after each mapping session, we incorporated feedback into the systems map. We also applied the mapping convention shapes to each portion of the map and condensed the text within the shapes to be more concrete. While there were initial challenges among experts understanding the adapted UML convention, we reviewed the mapping convention (Table 1) with each of the experts during sessions to ensure clarity, and adjusted the definitions as needed to foster a common understanding as we progressed with the mapping process.

Throughout this process, we consulted session notes to confirm that our edits between mapping sessions did not alter the intended meaning. We also consulted notes when there were discrepancies/inconsistencies among experts’ feedback. Our team collectively discussed how to reconcile these differences in terms of the relevance of the input to the scope of our map (e.g., we agreed to leave out factors beyond the scope such as potential solutions to overcome challenges and ways to mitigate climate change) and its applicability in communicating about climate change (e.g., we excluded inputs that pertained to climate change broadly, including attitudes toward climate change). We then discussed discrepancies with each expert during subsequent mapping sessions, reviewing the changes and rationale to collectively come to consensus on whether to keep/edit the inputs.

As part of this iterative process, we circulated each updated version of the systems map to the experts for them to review and send feedback to further refine the map (both prior to and after each session). The iterative process continued until our team reached a consensus with the experts that the systems map sufficiently represented the processes involved in climate change and its associated communication challenges.

## Results

### Map Overview

**Figure 1.**
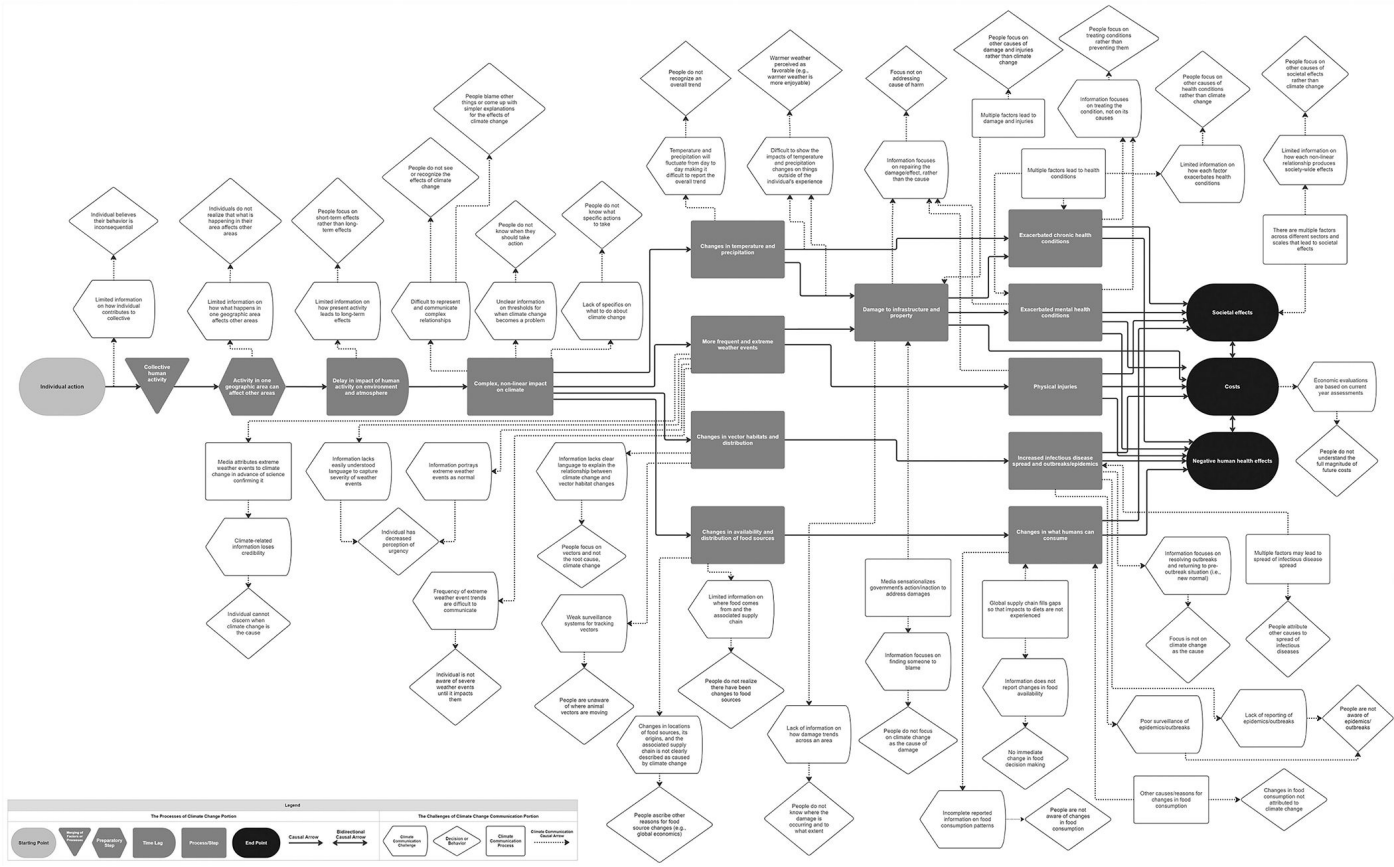
Systems map of the unique challenges to communicating climate-related information.

Figure 1 shows the resulting map. In the middle of the map, represented by gray and black shapes connected by solid arrows, is *The Processes of Climate Change* portion of the map, which depicts the major steps/processes linking human activity to climate to the resulting effects on the environment/atmosphere and human health. In *The Challenges of Climate Change Communication* portion of the systems map, emanating from each of these steps/processes (white shapes connected by dotted arrows) are challenges/issues in capturing and communicating information about each of these steps/processes (gray shapes) and how such challenges/issues then affect or result in human behaviors/decision-making (in diamond shapes) in ways that may prevent proper action. Table 2 lists the 27 different climate communication challenges identified by the map, and elaborates further on what each one represents with a description and example for each.

### Themes Identified in the Systems Map

Our map revealed seven major themes related to the challenges of communicating about climate change: (1) lack of data that quantifies relationships within the climate change process; (2) lack of integrated databases across multiple sectors; (3) difficulty communicating about processes that take time to evolve, emerge, and manifest; (4) lack of readily relatable and understandable climate lexicon; (5) the need to introduce climate-related concepts and language earlier in people’s lives and in the education system; (6) different disciplines that cover climate change-related processes work in silos; and (7) the need for new methodologies to describe the climate change process.

## Discussion

As indicated earlier, there is substantial evidence that current approaches to communicating the causes and effects of climate change have not been working. Determining how to change and improve such communication can be difficult due to the complexity involved. A systems map can help visualize and better understand and address such complexities and provide decision makers with a tool to use to address the challenges of climate change communication. For example, decision makers can use this systems map to understand where gaps in communication may exist, which communication approaches may be more impactful (e.g., are linked to multiple challenges), which should be prioritized, where more data collection may be most helpful, and how different communication challenges may have reverberating or indirect effects on the rest of the system. Ultimately, such a map can serve as a blueprint for systems models which can then be used to better design tailored and proactive communications about climate change (Lee et al., 2023; Lee, Bartsch, et al., 2017; Lee, Mueller, et al., 2017; Mabry et al., 2022). Further, our systems map helped identify many of the challenges in communication and revealed seven recurring themes.

One recurring theme is the lack of adequate data to characterize and quantify many of the key factors and processes. Thus, a priority would be to substantially increase the amount and type of data collected and improve its availability and presentation to the general public, the media, and other communication professionals. This would take more resources and greater investment (Rössler et al., 2019). One challenge is the data needed crosses multiple scales and scientific disciplines, which calls for greater collaboration across sectors and funding sources. It should be noted, however, that providing individuals with more knowledge alone does not necessarily lead to behavior change (Moser, 2010, 2016). Thus, targeted communication strategies will also be needed to impact behavior change.

A second recurring theme is the lack of databases that are integrated and shared across sectors, populations, and geographic locations (Gupta, 2007; Kirton & Warren, 2021). Since the processes involved in climate change cut across many different areas (Khanal et al., 2024), the lack of such databases prevents a more holistic, broader picture of what is happening and how different parts of the systems involved affect each other.

A third recurring theme is the difficulty in communicating about processes that take time to evolve, emerge, and manifest. Communication would have to overcome the tendency of people to focus much more on short-term, more immediate effects even when longer-term effects may ultimately have greater impact. An example is the news cycle that highlights a current event for a limited amount of time before it is supplanted by another current event (Jones, 2014). This is complicated by the fact that brief “breaking news” snippets have been replacing longer form and evergreen journalism on a number of media platforms. New emerging technologies and multimedia (Roosen et al., 2018) should be leveraged for a shift to a new paradigm where the media informs the public on the longer-term effects from climate change, not just acute, one-off events.

A fourth recurring issue is the dearth of lexicon/language that is more readily relatable to and understandable by the general public (Degeling & Koolen, 2022; Fenton, 2022; Moser, 2010). Many terms such as “100 Year Storm” may sound too technical, and at this point, is not an accurate description, and terms such as “global warming” may belie the dire effects of climate change for humans. This challenge is compounded by socio-cultural differences in how risk and information is perceived and acted upon (Adams, 2021; Marshall et al., 2019). A closer look at the psychological and sociological effects of different terms and developing a new lexicon that’s applicable across broader society can better facilitate communication (Appelgren & Jönsson, 2021; Harker-Schuch, 2019).

This raises a fifth recurring theme: the need to introduce the climate-change related concepts and language earlier in people’s lives and progression through the education system so that people are more familiar with them (Harker-Schuch, 2019). People may be more familiar with science fiction films’ portrayal of earth and the language used in their dialogs to describe earth’s systems rather than the concepts and terms used in climate science. For example, terms like industrial rain referenced in the popular sci-fi movie, *Blade Runner*, may be more familiar to the general public than climate science terms like ocean circulation, ocean acidification, and climate mitigation, even though these terms and concepts have greater relevance and real-world application.

A sixth recurring theme from the systems map is the siloing of different disciplines that cover climate change-related processes. As the map shows, these processes span many different areas ranging from meteorology to agriculture to different parts of the media (e.g., publications can arbitrarily separate journalists who cover flooding from those who cover the spread of infectious disease). Each can have a different entrenched culture, language, and approach to problem solving with potentially limited interactions due to historical rather than scientific reasons (Machalaba et al., 2015). This in turn can hinder collaboration and communication. Increased interaction, more shared language, and the emergence of hybrid individuals who cross disciplines and sectors can help break down such siloes.

Finally, the seventh theme identified is that there is a need for new methodologies to be used to describe and analyze the connections between human activity, climate change, and the impact on humanity as traditional methods alone have fallen short (Moser, 2010). Systems approaches can help public health decision makers, climate scientists, and climate change communication experts better understand the complex system that comprises communicating about climate change, including its global scope, heterogeneous patterns, complex causes, and wide-ranging impacts, and address these systems at many levels, ranging from the individual to the societal scale (Cox et al., 2021; Lee et al., 2023; Lee, Bartsch, et al., 2017; Mabry et al., 2022). Systems modeling has been used to study effective communication in other fields (Bruckmann, 1978; Fahey et al., 2003; Gillard & Johansen, 2004; Mekala et al., 2022), and these model frameworks may be adapted to climate change. One such example is the En-ROADS simulator (Climate Interactive, n.d..), which simulates the impact of various climate change policies on climate change and its factors/outcomes (e.g., energy prices, temperature, air quality, sea level rise).

Some of the root causes of these themes and challenges have been noted in previous work, such as climate change’s impacts spanning geographical locations and populations and difficulty communicating complexity (Leal Filho et al., 2019; Moser, 2010). However, our map provides a visualization of causal pathways and how they are related to each other in the system as a whole.

During the mapping process and subsequent interactions, the experts expressed that this method was new to them and they found its iterative process interesting, insightful, and engaging. They also noted that through interactive conversations they became more familiar with the method and could progressively see their feedback reflected in the map as it became more and more complex across sessions. Experts also expressed that this allowed for a process-driven training in systems mapping and more effective engagement between the team and the experts.

### Limitations

A systems map, like any conceptual model, is a simplification of real life and thus may not capture all factors/processes, effects, and elements. While our map attempts to identify most of the important factors involved in how climate change arises and impacts human health, some factors may have been missed. For example, we did not include feedback loops that may exist (e.g., changes in temperature/precipitation may change individual action), which are often present in complex systems and may offer potential intervention points in the system. Future iterations of the map could add such feedback loops. This map does not attempt to identify which links or relationships within the map may be more important than others, as they are all assigned equal weight. Also, our map intentionally aims to be generalizable across multiple populations, but contextual factors could influence the prioritization of factors/processes and their relationships in the map. Further, our map captures discussions with a limited number of climate change communication experts and may not capture all opinions on the topic. Since participatory systems mapping brings together the ideas of different experts, the resulting systems map may look different had different experts participated. It may have also looked different if it had been created in group sessions as discussions may lead to collective understanding and new ideas; however, multiple iterations meant experts saw the feedback/inputs from others and they were able to build upon and provide further input/feedback. Further, our systems mapping sessions did not include input from non-experts or the general public, thus it may be missing some perspectives. Future iterations of the map can include other experts and non-experts to account for additional perspectives. Lastly, there were certain theoretical ideas related to how communicating climate change is difficult (e.g., the politicization of climate change, climate fatalism leading to inaction) that arose in discussions with climate change communication experts. While these are important overarching challenges to communicating climate-related information, we focused the map on concrete communication challenges related to the specific processes involved in the causes and effects of climate change.

## Conclusion

Current approaches to communicating about climate change and its impacts on human health may be overlooking unique challenges. Through systems mapping, we can elucidate the factors and processes involved in climate communication, identify the different communication challenges, and develop enhanced approaches that more effectively communicate climate-related information. Such systems approaches are, in fact, essential to understanding and improving climate communication, otherwise new strategies may continue to overlook these challenges.

## Figures and Tables

**Table 1.** Systems mapping convention

Symbol	Description	What it Represents

**The Processes of Climate Change Portion of the Systems Map**		
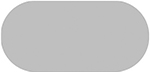	Entry/Starting Point	The start of the map and the climate change process
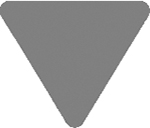	Merge	Two or more factors/processes merge together and become one at this junction
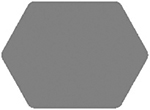	Preparation	A preparatory step that sets up another step in the climate change process
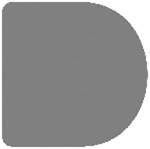	Delay	A time lag between the factors/processes
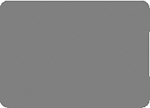	Climate Change Process	A process/step that occurs in the climate change process
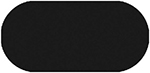	Exit/End Point	The end of the map
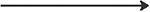	Causal Arrow	A causal relationship/pathway in the climate change process
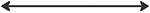	Bidirectional Arrow	An influential relationship between two factors/processes
**The Challenges of Climate Change Communication Portion of the Systems Map**		
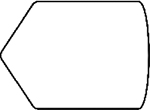	Display	Depicts climate change communication challenge/issue for a given process/factor/relationship in the climate change portion of the map
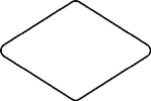	Decision/Behavior	The decision made/behavior exhibited by an individual that is affected by or is a result of the communication challenge
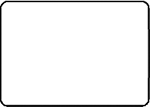	Climate Communication Process	A process/step that occurs in the climate communication process
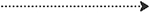	Climate Communication Causal Arrow	A causal relationship/pathway that shows where the climate communication challenge originates from and points to the associated display, decision/behavior, or climate communication process/step

**Table 2.** Climate communication challenges and their potential effects on human behavior and decision-making

Communication Challenge	Description	Example

Limited information on how individual actions contribute to collective human activity	Individuals often do not realize how their specific actions may contribute to the collective human activity that leads to climate change and thus, may not take steps to change their actions.	People may not realize that their individual action of driving to work adds to overall carbon emissions and that taking public transportation or carpooling are more environmentally-friendly options to lessen their impact.
Limited information on how what happens in one geographic area affects other areas	People in one geographic area may not realize how what happens in another geographic area may affect them.	Those in Brazil may not pay attention to carbon emissions in India, even though carbon emissions in both areas contribute to global climate change.
Limited information on how present activity leads to long-term effects	The time lag of climate change can make it difficult for an individual or organization to see the impacts of their actions. This can lead to a lack of awareness of how present activity leads to longer-term environmental effects.	Individuals may not realize that purchasing an electric vehicle leads to less carbon emissions over time, compared to gas or diesel vehicles.
Difficult to represent and communicate complex relationships	Due to the complex, non-linear impact that human activity has on the climate, it is difficult to communicate these relationships clearly. This can result in people not fully understanding the contributing factors to climate change and thereby potentially misidentifying causes.	Describing the relationship between greenhouse gas emissions and rising temperatures is not straightforward due to the complex processes involved. As a result, people may view rising temperatures as normal seasonal variations as opposed to the effect of excess atmospheric greenhouse gases trapping solar energy and warming earth’s temperatures.
Unclear information on thresholds for when climate change becomes a problem	Even when people understand a general relationship between human activity and a change in the climate, they may not realize the threshold at which that activity becomes a problem. As a result, it may not be clear when it is crucial to curtail or eliminate such activity.	While it may be understood that carbon emissions result in rising temperatures, people may not realize the point in time, or point at which a level of carbon concentrations in the atmosphere have been reached, where activities that produce carbon emissions, like airplane travel, are no longer viable. As a result, it may not be clear when it becomes critical to stop traveling by airplane.
Lack of specifics on what to do about climate change	Individuals may not be clear on what specific activities they can do to mitigate the effects of climate change because of the complex processes involved.	People may not be aware that eating less meat, especially beef and lamb (Our World in Data, (n.d.), and reducing dairy product consumption can reduce carbon emissions from transportation, preservation, and prolonged refrigeration (Fujimori et al., 2018).
Temperature and precipitation will fluctuate from day to day making it difficult to report the overall trend	Daily temperatures and precipitation vary from day-to-day and season-to-season. Individuals tend to focus on the day-to-day weather and may not notice larger sustained trends as a result of climate change.	Because temperatures tend to vary such that during the winter one day can be cold, the next might be warmer, and the following is cold again, one may not notice a rise in average winter temperature by 3.8 degrees (Raffa, 2023).
Difficult to show the impacts of temperature and precipitation changes on things outside of the individual’s experience	Without realizing more than the temporary effects of temperature (e.g., warmer weather) or precipitation changes (e.g., sunnier days) on daily activities and not the broader, longer-term effects, individuals may actually view such changes as favorable.	People may not realize that the same warmer weather that allows them to wear lighter clothes and spend time outdoors may also lead to decreased crop yields and limited availability of drinking water over time.
Climate-related information loses credibility	Media often attributes extreme weather events to climate change in advance of scientific experts (e.g., climatologists) confirming it. If it is determined that the event was due to some other natural occurrence and not climate change, individuals may question the attribution of climate change to different weather events and come to distrust climate science.	Media may jump to attribute climate change as the cause of a recent hurricane, but two weeks later, climatologists determine that it was not due to climate change, which leads to confusion and distrust among the public about attributing climate change to future events.
Information lacks easily understood language to capture the severity of weather events, portrays extreme weather events as normal, and the frequency of extreme weather event trends are difficult to communicate	Climate-related information tends to lack lay-friendly language that fully conveys the severity of extreme weather events, and the trends in their frequency of occurring. Additionally, the way extreme weather events are communicated may make these events seem more normal or typical than they are, thus, not adequately raising an alarm.	A forecast might show that it will be a “more active hurricane season” but obscures additional details about what this means in terms of severity or how this relates to previous years, or overall trends.
Information lacks clear language to explain the relationship between climate change and vector habitat changes	Climate information tends to lack clear language to connect climate change to the changes in locations of vector (e.g., mosquito, tick) habitats. As a result, individuals may think of climate change as having an impact on the weather they experience, but do not realize how this change in weather may lead to disease-carrying vector populations finding new habitat in their area.	Individuals may not realize that warmer weather and more rainfall led to disease carrying mosquitos moving in and finding habitat in these newly warmer and rainier areas. As such, individuals in those areas may focus on the elimination of mosquitoes without considering the cause of their movement stemming from climate change.
Weak surveillance systems for tracking vectors	Oftentimes the only way to know the arrival of disease-carrying vectors is through surveillance of new infections. Few current efforts proactively check the environment for the presence and extent of disease-carrying species (Wilson et al., 2020). This can lead to people being unaware of where vectors are moving and how they may be impacted by climate change.	The arrival of ticks to new areas that were previously uninhabitable for them may go unnoticed by those who live there due to a lack of surveillance systems to track their movement. As such, an increase in Lyme disease cases may be the only way to realize the change in tick habitat location to new areas, after the ticks have spread disease to human populations.
Changes in locations of food sources, its origins, and the associated supply chain is not clearly described as caused by climate change	Reports of changes in food sources tend to not include climate change as the root cause for the shift. As a result, individuals may ascribe other reasons for the changes. Additionally, most individuals in industrialized nations have little knowledge of where their food comes from. Thus, individuals may not realize changes in their food sources.	A blackberry distributor may notify consumers that their source location changed from Mexico to China but not include the reason for the change, such as climate change. As a result, individuals may ascribe other reasons for the change in location, such as the new location being cheaper or having better seasonality.
Information focuses on repairing the damage/effect, rather than the cause	When changes in temperature and/or precipitation and extreme weather events cause infrastructure and property damage, the outcome reports tend to focus on ways to repair the damages rather than noting climate change as the cause. Thus, individuals may focus on solely dealing with the resulting damages rather than the cause of the event itself. This difference tends to reinforce seeing extreme events as singular events rather than part of a growing trend.	Reports may focus on the costs of repair from a hurricane and federal assistance programs but neglect to note the role that climate change played with the hurricane.
Lack of information on how damage trends across an area	Reports of infrastructure damage tend to only report specific instances of damage, and not how damage trends across a geographic area.	Individuals may not realize how widespread property damage is beyond their immediate neighborhood, which obscures a full view of the magnitude of climate-induced damage.
Information focuses on finding someone to blame	When climate-related events cause infrastructure damage, media outlets tend to sensationalize the government’s action or inaction to address the damages. This leads to information that focuses on who or which entities are to blame rather than identifying climate change as the root cause of the problem.	After a severe flood leads to major property damage, the media may focus on reporting the lack of initiative from the government to repair the damages rather than noting the ways that climate change contributed to the damage.
Information focuses on treating the condition, not on its causes and Limited information on how each factor exacerbates health conditions	There are multiple factors that contribute to the development of chronic and mental health conditions, but there is limited information around the relationship between each factor and the resulting condition. Additionally, most information focuses on treating conditions rather than describing what may have caused them.	High air pollution exacerbates respiratory conditions, but the focus tends to be on treating the bodily effects (e.g., asthma inhalers) rather than mitigating or explaining the environmental impacts (e.g., burning fossil fuels) that contribute to these conditions.
Information focuses on resolving outbreaks and returning to pre-outbreak situation (i.e., new normal)	When an infectious disease outbreak occurs, the immediate focus tends to be on resolving the outbreak, in order to return to pre-outbreak conditions. This can result in individuals not realizing that the outbreak was potentially caused by climate change.	In the immediate aftermath of a disease outbreak, the focus of attention is typically on a “return to normal,” and may include activities like focusing resources on developing a vaccine. This may lead to a lack of awareness of the causes of the outbreak’s emergence, including the contribution of climate change.
Lack of reporting of epidemics/outbreaks and Poor surveillance of epidemics/ outbreaks	In general, there tends to be a lack of surveillance and reporting of infectious disease outbreaks until new infections emerge in populations. Further, when outbreaks become less severe (i.e., cases emerge less frequently) it may not be seen as a priority to continue surveillance efforts.	As mosquitos that carry Dengue virus are able to survive farther north, people may be unaware that Dengue outbreaks are occurring around them, resulting in a lack of protective measures for vulnerable populations, such as pregnant women.
Incomplete reported information on food consumption patterns and Information does not report changes in food availability	There lacks sufficient data on global food consumption patterns and food availability. Additionally, current data may be missing important details that climate researchers would need in order to track food changes on different geographic and demographic scales that might result from climate change.	Fluctuations in the availability of climate-resilient crops would have an impact on people’s health, however, it may not be apparent to researchers that this was happening until a more complete picture of their consumption patterns exists in retrospective data.
Limited information on how each non-linear relationship produces society-wide effects	Societal effects result from multiple factors across different sectors and scales, and people tend to assign greater weight to causes that are more immediate rather than climate change. As a result, individuals may not realize that climate change may be a contributing factor to the health effects they experience.	People tend to focus on more direct links to obesity, like poor diet choices, rather than causes that are less obvious or direct, like a lack of access to quality outdoor greenspaces for exercise in one’s neighborhood.
Economic evaluations are based on current year assessments	Economic evaluations that stretch over many years can be difficult for laypeople to interpret because they account for inflation and methodically reduce future costs. As a result, individuals may underestimate the true magnitude of future costs considerably. If it were more obvious how much climate change may cost in the future, people might be more willing to invest in solutions before a crisis occurs.	The current guidance that calls for a 3% or 7% discount rate on the social cost of carbon doesn’t adequately reflect the longer time periods of climate change (Prest et al., 2021). This may lead to an underestimation of the true costs of climate change and benefits of climate mitigation over time.

## References

[R1] Ackermann F, & Alexander J (2016). Researching complex projects: Using causal mapping to take a systems perspective. International Journal of Project Management, 34(6), 891–901. 10.1016/j.ijproman.2016.04.001

[R2] Adams M (2021). Critical psychologies and climate change. Curr Opin Psychol, 42, 13–18. 10.1016/j.copsyc.2021.01.007 33636522

[R3] Appelgren E, & Jönsson AM (2021). Engaging citizens for climate change—challenges for journalism. Digital Journalism, 9(6), 755–772. 10.1080/21670811.2020.1827965

[R4] Barbrook-Johnson P, & Penn A (2021). Participatory systems mapping for complex energy policy evaluation. Evaluation, 27(1), 57–79. 10.1177/1356389020976153

[R5] Barbrook-Johnson P, & Penn AS (2022). Participatory systems mapping. In Barbrook-Johnson P & Penn AS (Eds.), Systems Mapping: How to build and use causal models of systems (pp. 61–78). Springer International Publishing. 10.1007/978-3-031-01919-7_5

[R6] Bell A (2021). Sixty years of climate change warnings: The signs that were missed (and ignored). The long read. https://www.theguardian.com/science/2021/jul/05/sixty-years-of-climate-change-warnings-the-signs-that-were-missed-and-ignored

[R7] Bertscher A, Nobles J, Gilmore AB, Bondy K, van den Akker A, Dance S, Bloomfield M, & Zatoński M (2024). Building a systems map: Applying systems thinking to unhealthy commodity industry influence on public health policy. International Journal of Health Policy and Management, 13(1), 1–17. 10.34172/ijhpm.2024.7872 PMC11607592 39099529

[R8] Bruckmann CG (1978). A systems model of communication processes. Journal of Technical Writing and Communication, 8(4), 321–342. 10.2190/6yw6-4v0j-4prt-lcxy

[R9] Climate Interactive. (n.d.). The En-ROADS Climate Solutions Simulator. Retrieved May 14 from https://www.climateinteractive.org/en-roads/

[R10] Cox SN, Wedlock PT, Pallas SW, Mitgang EA, Yemeke TT, Bartsch SM, Abimbola T, Sigemund SS, Wallace A, Ozawa S, & Lee BY (2021). A systems map of the economic considerations for vaccination: Application to hard-to-reach populations. Vaccine, 39(46), 6796–6804. 10.1016/j.vaccine.2021.05.033 34045101 PMC8889938

[R11] Dabla-Norris E, Helbling T, Khalid S, Khan H, Magistretti G, Sollaci A, & Srinivasan K (2023). Public perceptions of climate mitigation policies: evidence from cross-country surveys. International Monetary Fund.

[R12] Degeling D, & Koolen R (2022). Communicating climate change to a local but diverse audience: On the positive impact of locality framing. Environmental Communication, 16(2), 243–261. 10.1080/17524032.2021.1998177

[R13] Fahey DK, Carson ER, Cramp DG, & Gray JAM (2003). Information communication technology in public health: The role of systems modelling. Health Informatics Journal, 9(3), 163–181. 10.1177/14604582030093004

[R14] Fenton D (2022, October 28). Climate change: A communications failure. The Hill. Retrieved May 10 from https://thehill.com/opinion/energy-environment/3709795-climate-change-a-communications-failure/

[R15] Fujimori S, Hasegawa T, Rogelj J, Su X, Havlik P, Krey V, Takahashi K, & Riahi K (2018). Inclusive climate change mitigation and food security policy under 1.5 °C climate goal. Environmental Research Letters, 13(7), 074033. 10.1088/1748-9326/aad0f7

[R16] Gillard S, & Johansen J (2004). Project management communication: A systems approach. Journal of Information Science, 30(1), 23–29. 10.1177/0165551504041675

[R17] Greenpeace. (2024). One year after Ohio disaster, Greenpeace USA is still calling on Biden administration to protect communities from hazardous plastics. https://www.greenpeace.org/usa/news/one-year-after-ohio-disaster-greenpeace-usa-is-still-calling-on-biden-administration-to-protect-communities-from-hazardous-plastics/

[R18] Gupta J (2007). The multi-level governance challenge of climate change. Environmental Sciences, 4(3), 131–137. 10.1080/15693430701742669

[R19] Harker-Schuch I (2019). Why is early adolescence so pivotal in the climate change communication and education arena? In Filho WL & Hemstock SL (Eds.), Climate Change and the Role of Education (pp. 279–290). Springer International Publishing. 10.1007/978-3-030-32898-6_16

[R20] Jones MD (2014). Communicating climate change: Are stories better than “just the facts”? Policy Studies Journal, 42(4), 644–673. 10.1111/psj.12072

[R21] Kallhauge AC (2023, December 13). COP28 Lays Foundation for Accelerated Climate Action with Much More Needing to Be Done. https://www.edf.org/media/cop28-lays-foundation-accelerated-climate-action-much-more-needing-be-done

[R22] Khanal S, Bolte G, Boeckmann M, & Males J (2024). Sector silos in climate action- a missed opportunity to prioritize health equity. PLOS Climate, 3(2), e0000349. 10.1371/journal.pclm.0000349

[R23] Kirton J, & Warren B (2021). From Silos to Synergies: G20 Governance of the SDGs, Climate Change & Digitalization. International Organisations Research Journal, 16(2), 20–54. 10.17323/1996-7845-2021-02-03

[R24] Kumarapeli P, De Lusignan S, Ellis T, & Jones B (2007). Using unified modelling language (UML) as a process-modelling technique for clinical-research process improvement. Medical Informatics and the Internet in Medicine, 32(1), 51–64. 10.1080/14639230601097705 17365645

[R25] Leal Filho W, Lackner B, & McGhie H (2019). An overview of the challenges in climate change communication across various audiences. In In (pp. 1–11). Springer International Publishing AG. 10.1007/978-3-319-98294-6_1

[R26] Lee BY, Bartsch SM, Mui Y, Haidari LA, Spiker ML, & Gittelsohn J (2017). A systems approach to obesity. Nutrition Reviews, 75(suppl 1), 94–106. 10.1093/nutrit/nuw049 28049754 PMC5207008

[R27] Lee BY, Greene D, Scannell SA, McLaughlin C, Martinez MF, Heneghan JL, Chin KL, Zheng X, Li R, Lindenfeld L, & Bartsch SM (2023). The need for Systems Approaches for Precision Communications in Public Health. Journal of Health Communication, 28(sup1), 13–24. 10.1080/10810730.2023.2220668 37390012 PMC10373800

[R28] Lee BY, Mueller LE, & Tilchin CG (2017). A systems approach to vaccine decision making. Vaccine: X, 35(1), A36–A42. 10.1016/j.vaccine.2016.11.033 PMC5460980 28017430

[R29] Lucidchart. (n.d.-a). Process Mapping Symbols and Notation. https://www.lucidchart.com/pages/process-mapping/process-map-symbols

[R30] Lucidchart. (n.d.-b). What Is Unified Modeling Language. https://www.lucidchart.com/pages/what-is-UML-unified-modeling-language

[R31] Luzi D, Pecoraro F, Tamburis O, O’Shea M, Larkin P, Berry J, & Brenner M (2019). Modelling collaboration of primary and secondary care for children with complex care needs: Long-term ventilation as an example. European Journal of Pediatrics, 178(6), 891–901. 10.1007/s00431-019-03367-y 30937604 PMC6511355

[R32] Mabry PL, Marcus SE, Clark PI, Leischow SJ, & Méndez D (2010). Systems science: A revolution in Public Health Policy Research. American Journal of Public Health, 100(7), 1161–1163. 10.2105/ajph.2010.198176 20530757 PMC2882409

[R33] Mabry PL, Pronk NP, Amos CI, Witte JS, Wedlock PT, Bartsch SM, & Lee BY (2022). Cancer systems epidemiology: Overcoming misconceptions and integrating systems approaches into cancer research. PloS Medicine, 19(6), e1004027. 10.1371/journal.pmed.1004027 35714096 PMC9205504

[R34] Machalaba C, Romanelli C, Stoett P, Baum SE, Bouley TA, Daszak P, & Karesh WB (2015). Climate Change and Health: Transcending Silos to Find Solutions. Annals of Global Health, 81(3), 445–458. 10.1016/j.aogh.2015.08.002 26615080 PMC7128244

[R35] Marshall NA, Thiault L, Beeden A, Beeden R, Benham C, Curnock MI, Diedrich A, Gurney GG, Jones L, Marshall PA, Nakamura N, & Pert P (2019). Our environmental value orientations influence how we respond to climate change. Frontiers in Psychology, 10, 938. 10.3389/fpsyg.2019.00938 31275184 PMC6591433

[R36] Mekala MS, Srivastava G, Park JH, & Jung H-Y (2022). An effective communication and computation model based on a hybridgraph-deeplearning approach for SIoT. Digital Communications and Networks, 8(6), 900–910. 10.1016/j.dcan.2022.07.004

[R37] Mincarone P, Leo CG, Trujillo-Martín MDM, Manson J, Guarino R, Ponzini G, & Sabina S (2018). Standardized languages and notations for graphical modelling of patient care processes: A systematic review. International Journal for Quality in Health Care, 30(3), 169–177. 10.1093/intqhc/mzx197 29346638

[R38] Moser SC (2010). Communicating climate change: History, challenges, process and future directions. WIREs Climate Change, 1(1), 31–53. 10.1002/wcc.11

[R39] Moser SC (2016). Reflections on climate change communication research and practice in the second decade of the 21st century: What more is there to say? WIREs Climate Change, 7(3), 345–369. 10.1002/wcc.403

[R40] Our World in Data. (n.d). Food: Greenhouse gas emissions across the supply chain. Retrieved March 19 from https://ourworldindata.org/grapher/food-emissions-supply-chain?country=Beef+%28beef+herd%29~Cheese~Poultry+Meat~Milk~Eggs~Rice~Pig+Meat~Peas~Bananas~Fish+%28farmed%29~Lamb+%26+Mutton~Beef+%28dairy+herd%29~Shrimps+%28farmed%29~Tofu~Coffee~Sunflower+Oil~Olive+Oil~Palm+Oil~Dark+Chocolate~Tomatoes

[R41] Pasquini G, Spencer A, Tyson A, & Funk C (2023). Why Some Americans Do Not See Urgency on Climate Change. Climate, Energy & Environment. https://www.pewresearch.org/science/2023/08/09/why-some-americans-do-not-see-urgency-on-climate-change/

[R42] Pecoraro F, & Luzi D (2022). Using unified modeling language to analyze business processes in the Delivery of Child Health Services. International Journal of Environmental Research and Public Health, 19(20), 13456. 10.3390/ijerph192013456 36294033 PMC9602458

[R43] Prest BC, Pizer W, & Newell RG (2021). Improving Discounting in the Social Cost of Carbon. Resources https://www.resources.org/archives/improving-discounting-in-the-social-cost-of-carbon/

[R44] Pronk NP, Eneli I, Economos CD, Bradley D, Fassbender J, Calancie L, Patawaran W, & Hovmand PS (2023). Using systems science for Strategic Planning of Obesity Prevention and Treatment: The roundtable on Obesity solutions experience. Current Problems in Cardiology, 48(8), 101240. 10.1016/j.cpcardiol.2022.101240 35513185

[R45] Raffa E (2023). Winter is here, but it’s losing its cool. CNN. https://www.cnn.com/2023/12/21/weather/us-winter-temperatures-climate-change/index.html

[R46] Roosen LJ, Klöckner CA, & Swim JK (2018). Visual art as a way to communicate climate change: A psychological perspective on climate change–related art. World Art, 8(1), 85–110. 10.1080/21500894.2017.1375002

[R47] Rössler O, Fischer AM, Huebener H, Maraun D, Benestad RE, Christodoulides P, Soares PMM, Cardoso RM, Pagé C, Kanamaru H, Kreienkamp F, & Vlachogiannis D (2019). Challenges to link climate change data provision and user needs: Perspective from the COST-action VALUE. International Journal of Climatology, 39(9), 3704–3716. 10.1002/joc.5060

[R48] Scott RJ, Cavana RY, & Cameron D (2016). Recent evidence on the effectiveness of group model building. European Journal of Operational Research, 249(3), 908–918. 10.1016/j.ejor.2015.06.078

[R49] Stickdorn M, Hormess M, Lawrence A, & Schneider J (2018). This Is Service Design Doing: Applying Service Design Thinking in the Real World (1st ed.). O’Reilly Media. https://www.thisisservicedesigndoing.com/methods/focus-groups

[R50] Tyson A, Funk C, & Kennedy B (2023). What the data says about Americans’ views of climate change. Environment & Climate. https://www.pewresearch.org/short-reads/2023/08/09/what-the-data-says-about-americans-views-of-climate-change/

[R51] United Nations. (n.d.). Climate Change. https://www.un.org/en/global-issues/climate-change

[R52] van den Akker A, Fabbri A, Alardah DI, Gilmore AB, & Rutter H (2023). The use of participatory systems mapping as a research method in the context of non-communicable diseases and risk factors: A scoping review. Health Research Policy and Systems, 21(1), 69. 10.1186/s12961-023-01020-7 37415182 PMC10327378

[R53] Vasilakis C, Lecnzarowicz D, & Lee C (2008). Application of Unified Modelling Language (UML) to the Modelling of Health Care Systems: An Introduction and Literature Survey. International Journal of Healthcare Information Systems and Informatics (IJHISI), 3(4), 39–52. 10.4018/jhisi.2008100103

[R54] Weise E (2023). Ipsos poll: 20% of Americans fear climate change could force them to move. The Nation. https://www.usatoday.com/story/news/nation/2023/09/06/climate-change-divides-america-usa-today-ipsos-poll-data-shows/70533243007/

[R55] Wilson AL, Courtenay O, Kelly-Hope LA, Scott TW, Takken W, Torr SJ, Lindsay SW, & Barrera R (2020). The importance of vector control for the control and elimination of vector-borne diseases. PloS Neglected Tropical Diseases, 14(1), e0007831. 10.1371/journal.pntd.0007831 31945061 PMC6964823

